# Evolution of adult male horn developmental phenotypes and character displacement in *Xylotrupes* beetles (Scarabaeidae)

**DOI:** 10.1002/ece3.7448

**Published:** 2021-03-25

**Authors:** Jen‐Pan Huang, Brett Morgan

**Affiliations:** ^1^ Biodiversity Research Center Academia Sinica Taipei Taiwan

**Keywords:** beetle horn, character displacement, evolutionary contingency, phylogenetic comparative method, *Xylotrupes*

## Abstract

Character displacement that leads to divergent phenotypes between sympatric species has been hypothesized to facilitate coexistence and promote the accumulation of biodiversity. However, there are alternative evolutionary mechanisms that may also lead to the evolution of phenotypic divergence between sympatric species; one of the mechanisms is evolutionary contingency. We studied the evolution of the presence and absence of a major male horn phenotype, which may have ecological implications for promoting coexistence between sympatric beetles, across geographic populations from different *Xylotrupes* beetles. By using a previously published phylogeny with 80 *Xylotrupes* taxa, we estimated the transition rates between the two phenotypic states (i.e., presence vs. absence of a major male phenotype). Based on the estimated transition rates, we then simulated possible phenotypic outcomes between sympatric species. We found that sympatric species were equally likely to evolve the same versus distinct phenotypic states based on the estimated transition rates given the phylogeny. The empirically observed number of sympatric species showing different phenotypic states can be explained by evolutionary contingency alone. We discussed the importance of applying phylogenetic comparative methods when studying phenotypic evolution and more generally to investigate the effect of stochastic processes before making deterministic inferences.

## Graphical abstract

Sympatric closely‐related species may exhibit different adaptive phenotypes, where character displacement is often invoked to explain such pattern. However, the evolution of distinct male horn phenotypes in sympatry can be explained by evolutionary contingency in Xylotrupes beetles. It is important to account for stochastic effect when study character evolution.

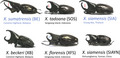

## 1 INTRODUCTION

**FIGURE 1:**
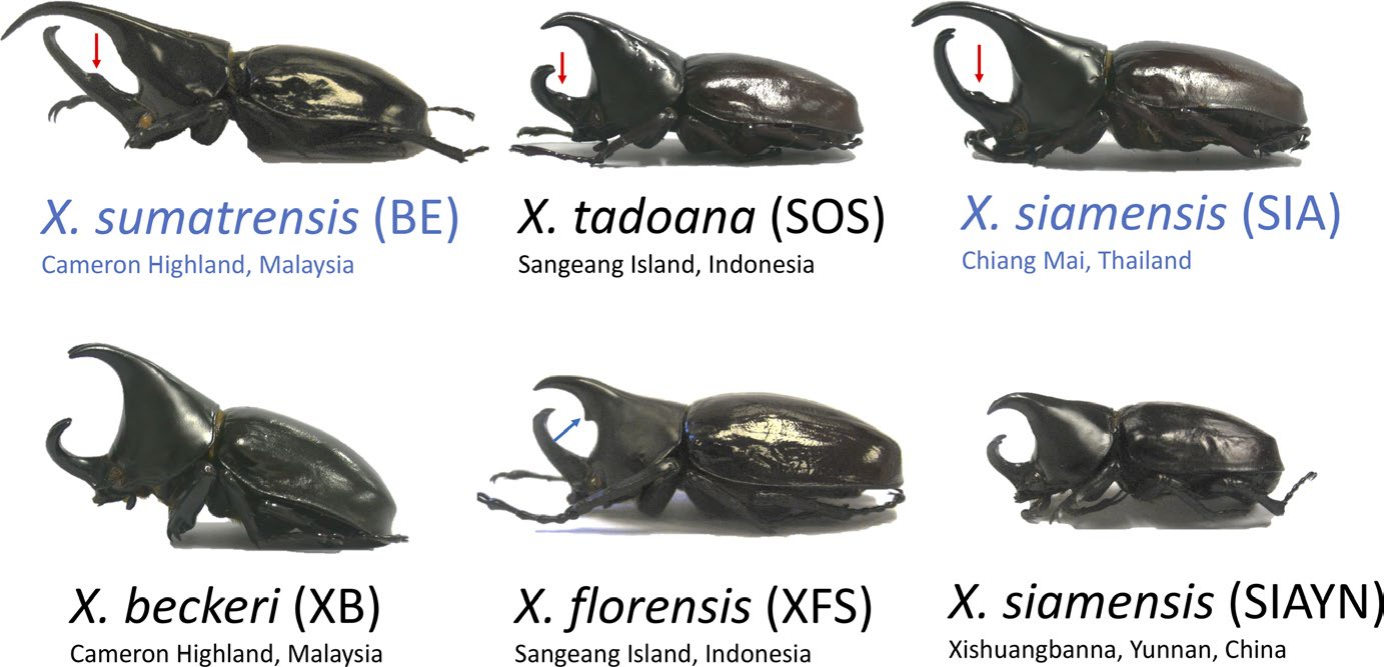
Examples of different adult male horn developmental phenotypes between distantly related sympatric (BE vs. XB; SOS vs. XFS) or between closely related allopatric (BE vs. SOS; SIA vs. SIAYN) *Xylotrupes* beetles. The population id of each examplar individual is in parentheses. Color arrows indicate denticles on cephalic (red) or thoracic (blue) horns. Species name in blue indicate species that have long‐horn male phenotypes

The causes and consequences of phenotypic evolution have been focused in evolutionary studies for their implications on how biodiversity may originate and accumulate because of and in response to different biological and ecological factors (e.g., Figure 1). One commonly studied phenomenon concerns phenotypic divergence between sympatric species that may facilitate coexistence, that is, character displacement (Losos, 2000; Schluter et al., 1985). Closely related species may share a similar niche preference because of their shared evolutionary history; when closely related species encounter each other in sympatry, such similar niche preference may lead to competitive exclusion unless one party evolves a different niche preference. Character displacement has been reported in many empirical cases, including the famous Darwin's finches (Grant & Grant, 2006; Schluter et al., 1985). The empirical studies always carefully examined alternative explanations that may have also affected phenotypic divergence between populations and species, and then made judgments whether character displacement occurred in the study systems and discussed how this evolutionary mechanism can facilitate coexistence (e.g., Kawano, 2002, 2003).

Except long‐term ecological observation studies that documented changes in phenotypes and the changes in different resource uses between sympatric species through time (Grant & Grant, 2006; Pritchard & Schluter, 2001), many empirical studies relied on comparing intraspecific differences across populations that were either sympatric or allopatric with respect to other closely related species (e.g., Kawano, 2002, 2003). The studies inferred character displacement if phenotypic divergence could only be observed when ecologically similar species were present in sympatry and when the intraspecific phenotypic difference could not be explained by geographic or ecological auto‐correlation alone (e.g., Kawano, 2002, 2003; Shapiro & Porter, 1989; Stuart & Losos, 2013). There are, however, other possible explanations, which could not be assessed by comparing phenotypic differences across populations alone, may also lead to phenotypic divergence between sympatric species (Stuart & Losos, 2013). One of such alternatives is evolutionary contingency where sympatric species evolved distinct phenotypes by chance (Gould, 1989; Losos, 1990; Stuart & Losos, 2013). When the pattern of character displacement seems apparent, yet exceptions may still exist, it could be difficult to empirically rule out the effect of evolutionary contingency with limited population sampling and limited information regarding the transition rates between phenotypic states. To statistically investigate the impact of chance on patterns of character displacement, one may need to approach the question via phylogenetic methods (e.g., Losos, 1990), where the transitions between phenotypic states are estimated and the results of phenotypic evolution because of stochastic character evolution evaluated.

**FIGURE 2:**
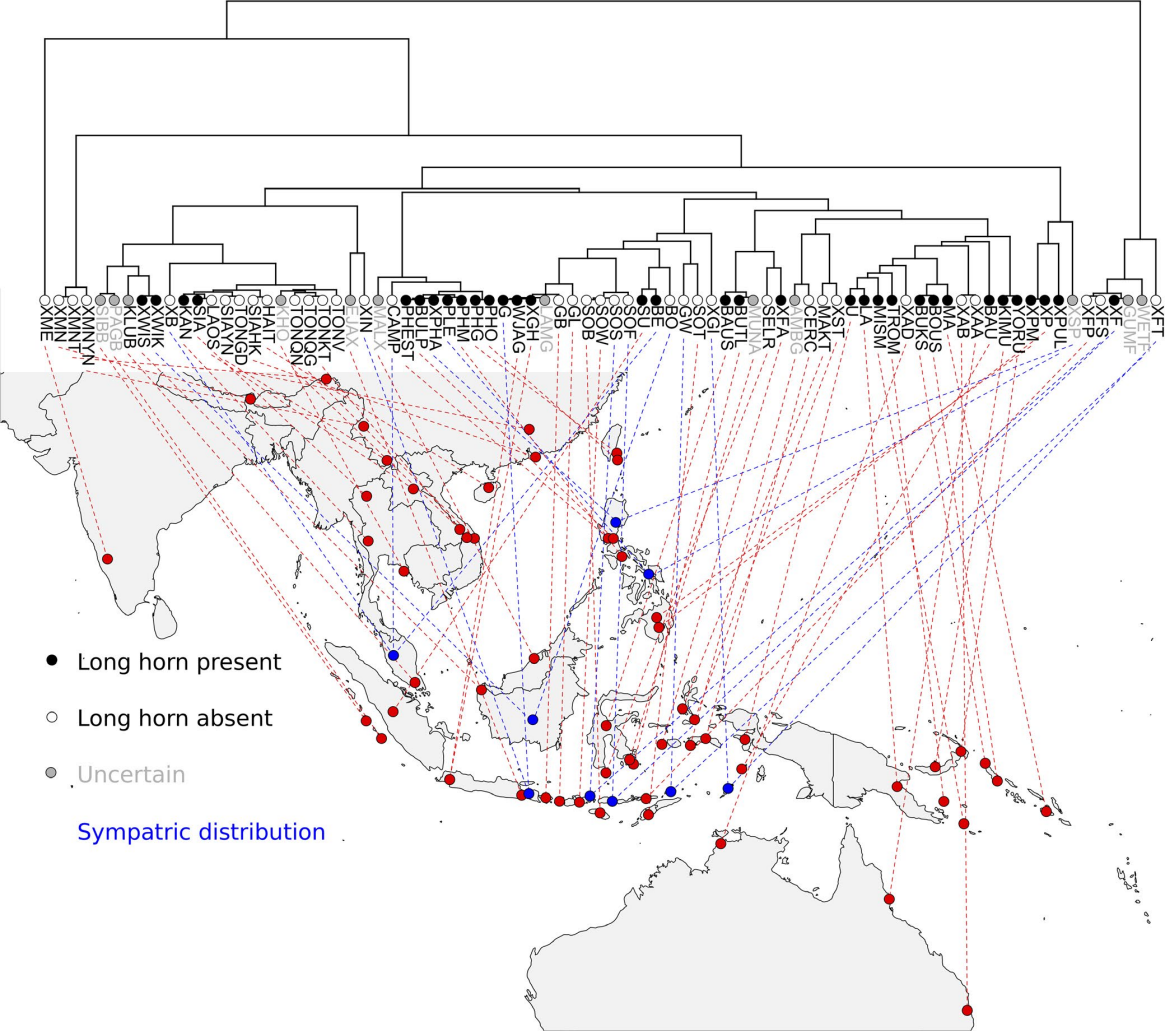
The population tree, geographic distribution (blue dots indicate sympatric distribution of multiple lineages), and adult male horn developmental phenotype (black or white dots on leaves of the population tree) of the studied *Xylotrupes* beetles. The Figure was made using the phylo.to.map function in phytools (Revell, 2012). The coordinate information was retrieved from a previous study (Huang & Knowles, 2018) and can be found in supplementary files

In the *Xylotrupes* beetle system, sympatric species pairs often exhibit different male horn developmental phenotypes (i.e., the presence or absence of a major male phenotype). A major male horn phenotype refers to a longer thoracic than cephalic horn (Taxa BE and SIA in Figure 1), while different male horn phenotypes may correspond to different reproductive tactics or fighting strategies (Emlen, 1997; Hongo, 2003). For example, major male phenotype can be found in the population of *X. florensis* from Flores island (Figure 1 and Hwang, 2011), where the sympatric *X. tadoana* population does not have the major male phenotype. However, such pattern does not always hold. For example, no major male phenotype can be observed on Sangeang Island for both *X. tadoana* and *X. florensis* (Figures 1 and 2). The evolution of major‐present and major‐absent phenotypes can be rapid and found between geographic populations of the same species (Rowland, 2011; Morgan & Huang, 2020). The evolution of different male horn phenotypes between sympatric species has been shown in other scarab beetle systems, where character displacement has been hypothesized as responsible for the observed pattern (Kawano, 2002, 2003). However, the support for character displacement happening in male horn phenotype in Giant Rhinoceros beetles was not as apparent as male genitalic phenotype (Kawano, 2002). The *Xylotrupes* beetle is therefore an ideal empirical system to test whether the evolution of major‐present versus major‐absent phenotypes between sympatric species may be the result of character displacement as hypothesized in a previous study focusing on *Chalcosoma* beetles (Kawano, 2002). Note that, body size range can be yet another aspect that may evolve rapidly between sympatric Rhinoceros beetles (Kawano, 2002), and the evolution of different body size can impact the threshold for the development of a major horn phenotype (Emlen, 1997; Moczek, 2003). However, body size range estimates require a large intrapopulation sampling size, which is currently not available for most *Xylotrupes* beetles (e.g., Rowland, 2003). Our focus on binary trait state was a simplification of the system, where the evolutionary diversification in male horn phenotype between sympatric species pairs may involve other factors that were not accounted for in the study (e.g., the evolution of different reaction norms according to body size). We employed a phylogenetic approach to study the phenotypic evolution with a specific focus on male horn phenotypes in *Xylotrupes* beetles, and based on the estimated evolutionary transition rates between phenotypic states we simulated possible outcomes of phenotypic evolution between sympatric species. We then discuss the implications of our study generally in relation to evolutionary determinism versus stochasticity, and more specifically about whether character displacement occurred in the *Xylotrupes* beetle system.

## 2 MATERIALS AND METHODS

### 2.1 Population tree and male horn developmental phenotypes

A population‐level species tree of *Xylotrupes* beetles (Figure 2) reconstructed using a multispecies coalescent model was retrieved from a previous publication that involved 80 geographic populations (Figures S1 and S2 in Huang & Knowles, 2018; the tree is provided as a supplementary material: XgPopTre.nex). The character state data set of male horn phenotypes for each taxon was derived from an illustrated book about Dynastine beetles of the world (Hwang, 2011). The taxonomy in the *Xylotrupes* section of the illustrated book followed the late *Xylotrupes* systematist, Dr. J. Mark Rowland, whose taxonomic expertise and personal collection also contributed to the previous *Xylotrupes* biogeographic study (Huang & Knowles, 2018). Additionally, the book not only showed images of male and female specimens from different species, but in some cases also from different populations of the same species (Hwang, 2011). Because the presence versus absence of a major male horn phenotype can evolve rapidly even between populations of the same species (Moczek, 2003; Morgan & Huang, 2020), the information about the male horn phenotype in different populations within species was crucial for this study. Conventionally, beetle‐illustrated books often showed major males of a species or a population, because of the impressive horn morphology. The absence of major male phenotype of a population in an illustration book possibly indicates the absence of major males in the population. Note that the absence of major male phenotype did not preclude the existence of such phenotype in a taxon; rather it only indicated that a major male phenotype has not yet been reported. Such bias may exist because of limited sampling information in terms of sample size and geographic range. We acknowledged our source of phenotypic data may be biased, and future studies that incorporate new data information are needed to investigate whether the result found in our study stands. The character state (0 for the absence and 1 for the existence of major horn phenotype) and the page number of the associated taxon from Hwang's illustration book can be found in the supplementary files (state_BAM.csv). Some of the taxa were not represented in the illustrated book; for those that we were uncertain whether the major male phenotype exists in the population, the state was coded as unknown (NA). The taxa with character state “NA” were removed when estimating the transition rates between states and reconstructing the ancestral state evolution (see below sections).

The *Xylotrupes* population‐level species tree was plotted onto a map using the phylo.to.map function in phytools (Revell, 2012) to visualize the geographic distribution of different taxa. The geographic distribution of each taxon was retrieved from Huang and Knowles (2018), and a sympatric distribution between taxa was called if two taxa from different species, or species complexes, coexist in geographic proximity, for example, they can be found in the same province or on the same island. Specifically, because *Xylotrupes* beetles have long been considered plant pests and have been shown to utilize many different host plant species (Firake et al., 2013), we assumed that all the species studied have a broad host preference and a similar habitat preference. Therefore, if two species have been recorded in geographic proximity, a sympatric distribution will be called.

### 2.2 Maximum likelihood estimation of male horn developmental phenotype evolution

**FIGURE 3:**
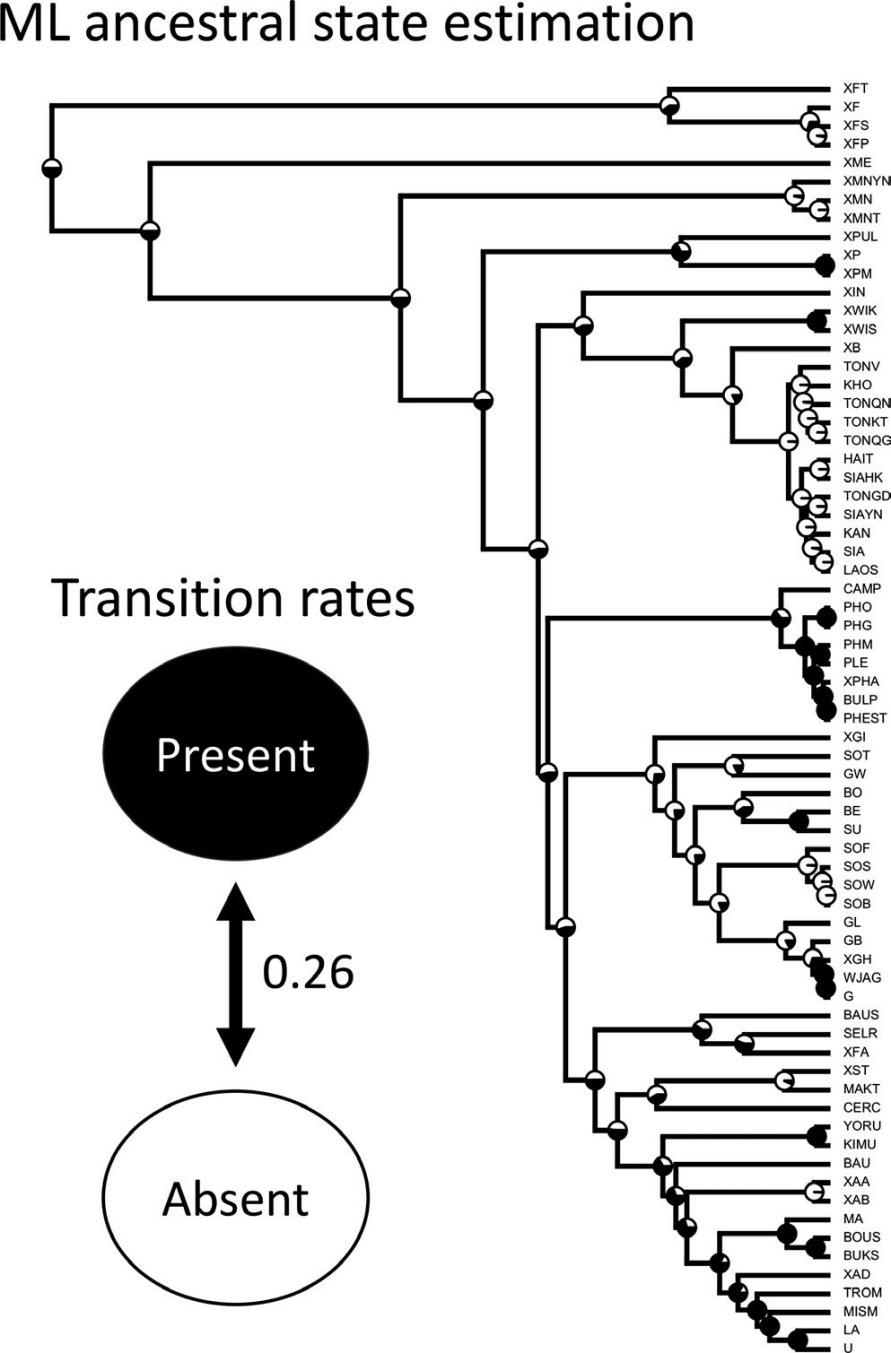
The maximum likelihood estimation of ancestral states and transition rates between states of adult male horn developmental phenotypes in *Xylotrupes* beetles

Twelve *Xylotrupes* taxa were excluded prior to estimating rates of transition between character states using the drop.tip function in ape (Paradis & Schliep, 2019) because we cannot confidently determine whether a major male phenotype exists in these taxa. The edited tree (68 taxa retained; Figure 3) and the trait file (0 and 1 for the absence and existence of a major male phenotype) were subsequently used to estimate the transition rates between character states and the ancestral character states on each node of the phylogeny using the ace function in ape (Paradis & Schliep, 2019). Specifically, we used both ER and ARD models in ace to estimate character state evolution. ER model assumes equal transition rates between states, while ARD allows transition rates to vary between states. We determined the goodness of fit for the two models using maximum likelihood and compared the two models using a chi‐square test function pchisq implemented in R. The ancestral states on nodes of the tree were then estimated using the transition rates from the selected model. We also performed a “total garbage test (Harmon, 2019)” to investigate whether there is some historical information when studying the major‐present and major‐absent states given our phylogenetic tree and whether the likelihood optimization techniques will work in our study case.

### 2.3 Simulating phenotypic evolution

**FIGURE 4:**
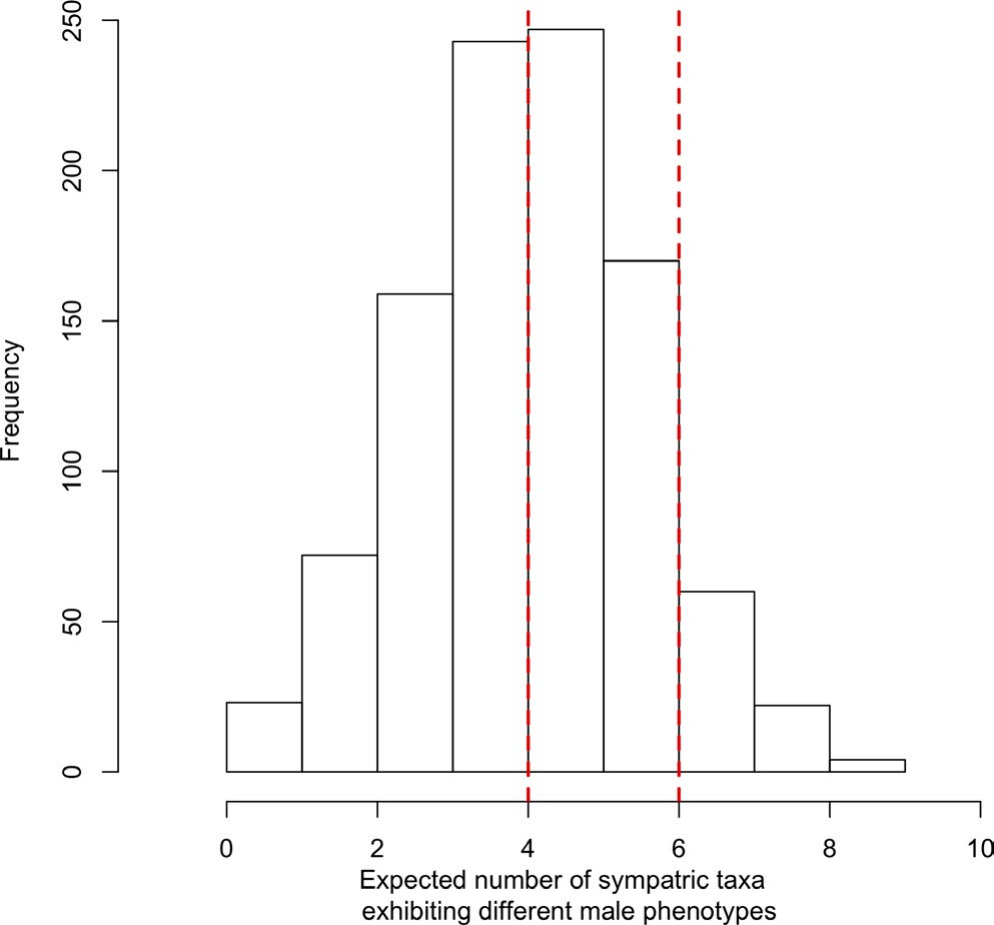
The expected number of sympatric species showing distinct male horn developmental phenotypes based on 1,000 simulations. The empirical accounts were shown using vertical dash red lines. Specifically, there were four confirmed cases of sympatric *Xylotrupes* species that exhibit distinct phenotypes; additionally, there were two uncertain cases where the phenotypic state for one or both species from sympatry cannot be determined. Therefore, the actual accounts of sympatric species with different phenotypes may range from 4 to 6

To estimate the odds of evolving a major‐present and a major‐absent species in sympatry, we used the *Xylotrupes* species tree (Figure 2; 80 tips) and the estimated transition rates between states from the previous section via the sim.history function implemented in phytools (Revell, 2012). Specifically, we counted the number of sympatric species pairs (blue dots in Figure 2) that differ in their character state after each simulation. A total of 1,000 data sets were simulated to generate an expected distribution of the number of sympatric species exhibiting different phenotypes based simply on the estimated transition rates. We then estimated the probability of observing different phenotypes between sympatric species based on our empirical observations (i.e., 4 or 6 cases out of a total of 9 sympatric localities). The empirical accounts for sympatric species with different male horn phenotypes were 4, while those with the same phenotype were 3. Because we were unable to determine the phenotypic state of *X. florensis* from Wetar Island (WETF), and *X*. sp from Luzon (XSP), the empirical accounts of sympatric species that evolve distinct phenotypic state can range from 4 to 6 (Figure 4). For each sympatric species pair, we also recorded the number of evolving different phenotypes between species from the 1,000 simulated data set.

## 3 RESULTS

There were nine instances of sympatric species pairs from the 80 studied taxa from the population‐level phylogeny of *Xylotrupes* beetles (Figure 2). Based on the binary character state and a modified phylogenetic tree that only included 68 tips (Figure 3), the log likelihood values calculated using ER and ARD models for trait evolution were −35.1983 and −34.96077, respectively. Although the ARD model gave a better likelihood, the difference was not statistically significant (*p* = .490672). The garbage model resulted in a terrible fit to our data (lnL = −47.64538), which is much worse than those of ER and ARD models. Therefore, we used results from the ER model for ancestral state estimation (Figure 3). The estimated transition rate between the two character states was 0.26 (standard error = 0.0889) based on the ER model. The estimated transition rates based on ARD model were 0.3007 ± 10.1058 (SE) and 0.2351 ± 0.0844 (SE) from major‐present to major‐absent and from major‐absent to major‐present, respectively. The estimated ancestral states at deeper nodes in the phylogeny were highly uncertain. Recently diverged lineages often shared the same phenotype where the estimated ancestral state of their common ancestor highly likely had the same phenotypic state.

Based on 1,000 simulations of character evolution using the estimated transition rates between phenotypic states, all the pairs of sympatric taxa had roughly a 50% chance of evolving different phenotypes (data not shown but can be reproduced with the supplementary R code). Furthermore, the simulated distribution of the number of sympatric taxa that evolved different phenotypes peaked around 4 and 5 out of 9 possible instances (Figure 4). The probability of evolving 6 or more accounts of different sympatric male horn phenotypic states based our simulations was 256 out of 1,000. The number of simulations that resulted in 7, 8, and 9 accounts of a major‐present and a major‐absent sympatric species were 60, 24, and 2, respectively. Our results thus indicated that the effect of evolutionary contingency in generating a major‐present and a major‐absent species in sympatric *Xylotrupes* beetles cannot be statistically rejected.

## 4 DISCUSSION

The evolution of distinct phenotypic states in traits that may have evolutionary or adaptive significance between closely related sympatric species has long been an example for niche partitioning that facilitates coexistence (Losos, 2000). However, although many studies have attempted to test deterministic explanations for character displacement (e.g., Kawano, 2002, 2003), few studies have considered the effect of evolutionary contingency (Losos, 2011), where the observed pattern of evolving distinct phenotypes between sympatric species may result because of stochastic character evolution. We showed that in *Xylotrupes* beetles the rapid transition between the presence and absence of a major male horn phenotype among taxa (populations and/or species) can frequently result in sympatric species pairs that exhibit different phenotypic states. Note that we are not downplaying the importance of character displacement and competition nor species interaction in promoting phenotypic divergence and coexistence; instead, our results aim to bring an often neglected evolutionary force, that is, stochasticity and contingency, into the discussion when studying character evolution using phylogenetic comparative methods.

The implications of our results extend beyond character displacement. For example, a common and strong selection pressure may lead to phenotypic convergence between sympatric species in cases of mimicry and camouflage (e.g., Buckley et al., 2008). The pattern of phenotypic convergence may however also arise via evolutionary contingency or stochasticity (Losos, 2011). A previous empirical study has shown that both deterministic and contingent mechanisms have contributed to the body shape convergence in *Lerista* lizards (Morinaga & Bergmann, 2017). On the other hand, a study focusing on the imperfect Müllerian mimicry in a Chilean ground beetle complex has statistically eliminated the effect of evolutionary contingency on generating phenotypic convergence between sympatric species (Muñoz‐Ramírez et al., 2016). Nevertheless, while the selection pressure might be strong enough to promote convergence in aposematic body coloration, they hypothesized that demographic histories, yet another evolutionary force of stochasticity (e.g., Huang et al., 2018), may have impacted the pattern of imperfect mimicry among *Ceroglossus* species. Phenotypic change according to different habitat types or facing different biological interactions have often been hypothesized of adaptive and/or deterministic origins (Gould & Lewontin, 1979; Nielsen, 2009), but random stochastic process and evolutionary contingency that may lead to similar patterns should not be neglected.

Our results demonstrated the importance of studying character evolution and divergence using phylogenetic methods, where multiple populations per species were included for estimating the character evolutionary history. We showed that a model assuming asymmetric transition rates between the absence and presence of a major phenotype (ARD) did not fit the empirical data statistically better than another one assuming equal transition rate (ER), implying that the transition rate could be treated as the same between the two directions. Additionally, the ancestral states at deeper nodes could not be effectively estimated without uncertainty (Figure 3). For example, the ancestral states at deeper nodes were equally likely for either major‐present or major‐absent phenotypes; on the other hand, estimated phenotypic states for nodes leading to recently diversified taxa often had higher probabilities favoring one over the other states (Figure 3). Furthermore, by simulating character evolution based on the estimated transition rates, the *Xylotrupes* phylogeny could result in approximately the same occurrences of evolving a major‐present and a major‐absent sympatric species as observed empirically (Figure 4). As mentioned before, conventional investigations for character displacement studies often focused on comparing the similarities and differences in phenotypic traits across sympatric and allopatric populations both within a species and between different species (Kawano, 2002, 2003; Losos, 2000; Newman & Anderson, 2020; Schluter et al., 1985). While confounding effects such as spatial or ecological auto‐correlation of phenotypic states can indeed be eliminated by comparing and contrasting character states across populations and between species, an alternative explanation where rapid phenotypic evolution can result in divergent phenotypes between sympatric species could not be ruled out. Using a phylogenetic approach, we showed that a seemingly character displacement pattern in *Xylotrupes* beetles can result because of evolutionary contingency.

Character displacement may nevertheless still be an important mechanism in promoting coexistence between *Xylotrupes* beetles, even though our results demonstrated that evolutionary contingency alone can explain the evolution of difference in the presence and absence of a major male phenotype in sympatry. Previous studies have shown that the pattern of character displacement was more apparent in genitalic structure, but less so in male horn structures in the Giant Rhinoceros beetle, genus *Chalcosoma* (Kawano, 2002). Since male horn structure is for male–male competition but not for mate recognition nor for resource usage, one may not expect strong selection force being imposed on this trait comparing to other traits of reproductive and survival significances (e.g., the genitalia; Kawano, 2002, 2003). Because we only studied the evolution of major‐present and major‐absent phenotypes, we cannot rule out the possibility that character displacement may have occurred in other traits. Furthermore, although some sympatric *Xylotrupes* species are both major‐absent, they often have different horn structures or shapes. For example, *X. tadoana* (SOS) and *X. florensis* (XFS) can be found on Sangeang Island, where a major male phenotype is absent in both taxa (Figure 1). The taxon SOS has a specific cephalic horn denticle and XFS a thoracic horn denticle that the two sympatric taxa can be easily distinguished. There are additional species‐specific traits that can be used to distinguish sympatric species, such as the pubescence on elytra and pronotum that separate *X. pubescens* from its sympatric *X. philippinensis* (Silvestre, 2006). The functions of horn denticles and elytral pubescence have not yet been studied, but they can be phylogenetically conservative. The elytral pubescence can only be found in *X. pubescens* (Hwang, 2011; Silvestre, 2006), the thoracic horn denticle only occurs in *X. florensis* (Hwang, 2011), and the cephalic denticle exists mainly in the *X. gideon* species group and some localized populations of *X. siamensis* (Huang & Knowles, 2018; Hwang, 2011; Morgan & Huang, 2020).

We would like to emphasize that whether we can observe a major‐present and a major‐absent *Xylotrupes* species in sympatry may have geographic structure, where sympatric species from Sundaland, that is, Java, Malay Peninsula, and Borneo, always exhibit distinct phenotypic states (Figure 2; Silvestre, 2004). On the other hand, sympatric species from the Philippines often share similar male horn shape and size, but as mentioned in the previous section they can easily be distinguished by the presence or absence of pubescence (Silvestre, 2006). Sympatric species pairs from Wallacea are found between distantly related lineages (Huang & Knowles, 2018), where species from one of the lineages, *X. florensis*, exhibits a distinct thoracic denticle that distinguishes males from this species from all the other *Xylotrupes* beetles (Figure 1); different sympatric species may either share the same (e.g., between SOS and XFS from Sangeang Island; Figures 1 and 2) or have different phenotypic states (e.g., between XF and SOF from Flores Island; Figure 2). Furthermore, a smaller island size might lead to smaller body sizes of the inhabiting beetle species (island rule; see Lokatis & Jeschke, 2018), where a major male may evolve less frequently (Emlen, 1997; Moczek, 2003). For example, two pairs of sympatric taxa without a major male phenotype are from two small islands (Wetar and Tanimbar islands), although exception also exists where a major male phenotype did evolve in a small island taxon from the island Flores (taxon XF). Our current incomplete sampling effort, albeit large and extensive geographic sampling, is however incomprehensive at the population level and thus prevents us from testing the evolutionary nuances, such as geography, habitat/island size, and the process and stage of speciation (Huang, 2020), that may affect phenotypic evolution and lead to character displacement. Future comparative studies that focus on fine spatial scale population sampling across multiple species using many traits of putative adaptive functions are needed to test whether character displacement may have occurred between sympatric *Xylotrupes* beetles and under what geographic, ecological, or evolutionary conditions will we expect to observe character displacement in certain traits.

## CONFLICT OF INTEREST

The authors declare there is no conflict of interest.

## AUTHOR CONTRIBUTIONS

**Jen‐Pan Huang:** Conceptualization (lead); Data curation (lead); Formal analysis (lead); Funding acquisition (lead); Investigation (lead); Methodology (lead); Project administration (equal); Resources (equal); Software (lead); Supervision (lead); Validation (lead); Visualization (lead); Writing‐original draft (lead); Writing‐review & editing (lead). **Brett Morgan:** Data curation (equal); Formal analysis (supporting); Methodology (supporting); Resources (supporting); Visualization (supporting); Writing‐review & editing (supporting).

## Data Availability

The R code for all the analyses and plots, input tree file, and the character state file can be downloaded from https://github.com/airbugs/xylotrupes_CharDis.
